# Exclusive Breastfeeding Rates Among Roma and Non-Roma Mothers in Greece: A Single-Center Cross-Sectional Study from “Tzaneio” General Hospital of Piraeus

**DOI:** 10.3390/clinpract14060185

**Published:** 2024-11-04

**Authors:** Artemisia Kokkinari, Evangelia Antoniou, Eirini Orovou, Maria Tzitiridou-Chatzopoulou, Maria Dagla, Georgios Iatrakis

**Affiliations:** 1 Department of Midwifery, School of Health & Care Sciences, University of West Attica, 12243 Athens, Greece; lilanton@uniwa.gr (E.A.); mariadagla@uniwa.gr (M.D.); giatrakis@uniwa.gr (G.I.); 2 Department of Midwifery, School of Health & Care Sciences, University of Western Macedonia, 54636 Kozani, Greece; eorovou@uowm.gr (E.O.); mtzitiridou@uowm.gr (M.T.-C.)

**Keywords:** Roma, gypsy, travellers, exclusive breastfeeding

## Abstract

Background: Exclusive breastfeeding is vital for the optimal development of infants, yet the practice of using infant formula has become increasingly prevalent. While many studies globally investigate factors affecting breastfeeding, there is a scarcity of research focusing on marginalized groups, particularly the Roma community. This study seeks to compare the breastfeeding rates of Roma and non-Roma mothers upon discharge from a maternity hospital in Greece. It also examines factors contributing to the decline in breastfeeding among Roma women, with particular emphasis on the role of midwifery support. The aim of this study is to promote the development of supportive policies and programs for breastfeeding among Roma mothers. Methods: A cross-sectional study was conducted from September 2019 to January 2022, involving 248 infants born at ≥37 weeks of gestation and their mothers, who were of Greek nationality. Both Roma and non-Roma participants received consistent, high-quality care from the same midwife researcher, who personally attended to them. All participants initiated breastfeeding immediately after their newborns’ births and practiced rooming-in by keeping their babies in the room with them during their hospital stay. Data were collected through questionnaires to determine the rates of exclusive breastfeeding among the two groups. Results: The study found that a smaller proportion of Roma mothers exclusively breastfed their infants compared to non-Roma mothers, despite receiving similar levels of support from healthcare professionals. Conclusions: The provision of midwifery support did not significantly enhance exclusive breastfeeding rates among Roma mothers. This suggests the need for more comprehensive and multi-faceted interventions. Further research is required to confirm these findings and to design effective programs aimed at increasing exclusive breastfeeding rates, thereby improving health outcomes for Roma children and mothers.

## 1. Introduction

Breastfeeding is the most natural and beneficial way to nourish infants, offering significant advantages for both children and mothers. The World Health Organization (WHO) recommends exclusive breastfeeding for the first six months of life, as it provides all the essential nutrients needed for growth and health. Beyond six months of age, breastfeeding should continue alongside complementary foods up to two years of age or more, depending on the preferences of the mother and child [1]. A systematic review by Kramer and Kakuma [2] supports these guidelines, demonstrating that exclusive breastfeeding for six months provides more benefits than breastfeeding for 3–4 months followed by mixed feeding. The benefits of breastfeeding are numerous and well-established. It can prevent more than 820,000 child deaths annually, particularly in low- and middle-income countries, and reduce the risk of breast and ovarian cancers and type 2 diabetes, potentially preventing around 20,000 maternal deaths each year [3].

Despite these benefits, the global rates of exclusive breastfeeding remain inadequate. Only 42% of newborns are breastfed within the first hour of life, and just 41% of infants under six months are exclusively breastfed [4]. These rates vary widely, with higher-income countries typically reporting lower rates, while traditional practices in low- and middle-income countries often lead to higher rates. WHO assessments across 84 countries highlight a widespread lack of policy support for breastfeeding at the national level [5]. Ensuring accessible and high-quality breastfeeding support for all women is a crucial social investment. Breastfeeding disparities indicate that socioeconomically vulnerable women, including those from marginalized ethnic or racial groups, encounter significant barriers to breastfeeding as recommended or desired [6]. Addressing these inequities is vital, as the public health benefits of breastfeeding can significantly reduce healthcare costs. Therefore, equitable access to breastfeeding programs, supported by evidence-based policies and infrastructure that promote, protect, and support breastfeeding, should be considered a matter of social justice [6]. Any social, economic, legal, political, or biomedical factor preventing women from exercising their right to breastfeed should be framed as a health inequity, a social injustice, and ultimately a human rights violation [6].

The Gypsy, Roma, and Traveller communities in the UK consist of various cultures and ethnicities. The largest of these groups is the Romani, which comprises English and Welsh Gypsies and European Roma communities. Additionally, there are Irish and Scottish Travellers, Travelling Entertainers (fairground and circus workers), Boat Dwellers, and New Age Travellers. The term ‘Gypsy’ should be used with caution. While English and Welsh Romani communities generally accept the term ‘Gypsy’ and use it to self identify, it is considered a racial slur by many European Roma communities, making its use deeply inappropriate to describe them. Although many people refer to Gypsies, Roma, and Travellers as a single, homogeneous group, cultural practices can vary widely among the different groups and even within communities. Infant feeding culture is no exception [7].

While international data, including those from the UK, highlight similar challenges faced by minority communities, this study specifically examines the situation of Roma women in Greece. The Roma represent one of the largest and most marginalized minority groups in Greece, facing significant socioeconomic difficulties. Notably, the Roma population exhibits a high concentration of young individuals, a moderate representation in the working-age group, and a virtually nonexistent elderly population. Only 1.6% of Roma individuals are over 65 years old, compared to 16.7% in the general population. The average age of the Greek Roma population is estimated to be 21.56 years, compared to 42.2 years for the general Greek population [8]. Furthermore, the child dependency rate is 43.39% for the Roma, compared to 15.69% across Europe. Although official statistics on the Roma are limited, it is estimated that women of reproductive age (ages 10 to 14: 7%, 15 to 19: 5.5%, 20 to 24: 5%, 25 to 29: 5%, 30 to 34: 4.5%, and 35 to 39: 3%) account for approximately 30% of the total Roma population, according to a study on national policies [8].

The Roma community in Greece has a long-established presence, preserving its traditions while encountering barriers to education and healthcare services. The total proportion of the Roma within the overall Greek population is estimated to be between 2 and 3%. Estimates of the size of the Greek Roma population range from 180,000 to 365,000 people, with an average estimation of around 270,000 being more reflective of reality. Significant concentrations of Roma families (over 1000) are located in four regions: Eastern Macedonia-Thrace, Thessaly, Western Greece, and Central Macedonia. There is a noticeable spatial concentration of Roma in specific areas, neighborhoods, suburbs, or villages. This concentration often results in social disintegration and reinforces their social exclusion, particularly concerning Roma women’s access to justice [9]. Regarding their demographic characteristics, data reveal that the age structure and age categories of the Roma population significantly differ from those of the general Greek population. Specifically, the Roma population shows a high concentration of young individuals, a moderate to low concentration in the productive age groups, and a nearly nonexistent elderly population [8].

Roma individuals are known to have a poor health status and limited access to health services, even compared to other ethnic minority groups. Members of this marginalized ethnic group are rarely consulted about their health needs or health service provision. The majority of the Roma population, especially among older age groups, remains illiterate. While school attendance is more prevalent among younger Roma than among their older peers, their engagement in the educational system is still considered insufficient to improve their vocational status and mobility. Most Roma children aged 12 and older drop out of school to seek employment and contribute to the family income. The health issues faced by the Roma population are closely linked to their low socioeconomic status, inadequate living and working conditions, and limited educational attainment. These factors contribute to higher rates of morbidity, poor health, reduced life expectancy, and increased child mortality [9].

Optimal infant feeding in the first year of life can significantly enhance lifelong health [10]. Roma are recognized as one of the most disadvantaged minority groups both in the United Kingdom and worldwide. Research into the health needs of this group is an emerging field, and Roma can justly be described as an “invisible minority” [10]. Access to health services for Roma is also known to be inadequate [10]. Roma have a history of persecution and rejection by mainstream society, which persists today [10].

Small-scale studies indicate that breastfeeding rates among Roma mothers have significantly declined [7]. Pinkey et al. [11] note that breastfeeding initiation rates among English and Welsh Roma, as well as Scottish and Irish Travellers, were as low as 3%, with none of the Roma mothers continuing to breastfeed six weeks postpartum. It is reasonable to assume that the decline in breastfeeding rates coincided with the normalization of infant formula feeding within the British working class around the mid-20th century [7]. Today, formula feeding is largely seen as the cultural norm among the Roma [7]. However, breastfeeding rates are significantly higher in European Roma communities, where breastfeeding is culturally normative, with some Roma mothers describing the practice as integral to their cultural identity [7,10]. Nonetheless, the cultural shift from breastfeeding to bottle-feeding continues to this day [7].

Generally, Roma mothers encounter additional challenges due to a lack of adequate support [7]. Some Roma communities demonstrate higher rates of illiteracy. However, even when a mother indicates her inability to read, written informational pamphlets are still provided as sources of breastfeeding information [7,10]. For European Roma mothers, this lack of adequate support is often offset by the backing of their family and community [7]. However, as breastfeeding knowledge and support diminish within their communities, Roma women often seek external support [7,10].

A prevailing perception exists that Roma mothers often trust the advice and suggestions of their community and family members more than information and recommendations from external professionals [7]. They believe that a mother who has birthed and raised many children is often seen as a better source of information than a midwife or health visitor [7].

In contrast to the above findings, the literature review showed limited research supporting the claim that this ethnic group is increasingly abandoning breastfeeding. In fact, only one study [12] confirms this trend, while practical observations in healthcare settings suggest that Roma women are more likely to adopt formula feeding to align with their current lifestyles. Roma mothers now believe that infant feeding has undergone changes in fashion over time; currently, the trend favors breastfeeding, despite the availability of infant formula milk similar to human milk, which supports excellent infant development and facilitates shared infant care [12]. This perspective may seem surprising in a community where mothers heavily influence their daughters’ decision-making. However, it is important to remember that today’s Roma grandmothers often experienced the early cessation of breastfeeding or avoided it altogether, and consequently integrated formula milk into their feeding practices. This feeding pattern is transmitted to their daughters, who fully trust and value their mothers’ knowledge, seniority, and experience [12]. Bureaucratic processes in health systems, discrimination, negative attitudes among some healthcare personnel, language barriers, cultural differences, economic hardship, and characteristics of these minorities, such as a fear and mistrust of the health system and healthcare professionals, create even greater barriers [13]. Roma very often face common issues regarding barriers to accessing health services.

Midwives have a critical responsibility to uphold respect, dignity, and fairness while actively addressing discrimination and advocating for vulnerable individuals. These responsibilities and reinforced commitments, supported by the Nursing and Midwifery Council’s (NMC) Standards of Proficiency for Specialist Community Public Health Nurses (2022) and the NMC Code (2018), are designed to reduce inequality and improve health outcomes universally [14].

This study aimed to examine the prevalence of exclusively breastfed infants on the third day of life, before discharge from the maternity ward, in both non-Roma and Roma populations, as well as the factors influencing this practice. The research was carried out at Tzaneio General Hospital of Piraeus, where a consistent and standardized support system was provided by the midwives staffing the facility.

## 2. Materials and Methods

This cross-sectional study was conducted from September 2019 to January 2022 and included 248 healthy mother–newborn pairs. Participants were pregnant Greek women with a gestational age of 37 weeks or more, who gave birth to a single, live infant at the hospital’s Obstetrics and Gynecology department. Women who were not of Greek nationality, those who gave birth before 37 weeks of gestation, had multiple births, or experienced pregnancy complications or maternal/fetal health issues were excluded from the study. The aim was to assess the rate of exclusive breastfeeding among these pairs, with a focus on the differences between the Roma and non-Roma populations.

Comprehensive personal questionnaires were administered to document the characteristics of each mother and newborn, supplemented by data from their medical records. The newborns’ physical measurements were taken immediately after birth, with birth weight recorded as the average of three measurements using a calibrated scale. To ensure accuracy, newborns were weighed within one hour of birth, with all measurements performed by midwives. Additional factors influencing exclusive breastfeeding were identified through a thorough review of medical histories and responses to personal questionnaires.

The study was designed to explore the rate of exclusive breastfeeding at three days postpartum in both non-Roma and Roma populations, along with the factors that may influence this practice. The goal was to determine whether there are differences in breastfeeding patterns between these groups. Should any differences be found, it would suggest that midwives should tailor their support to better meet the needs of these mothers.

The study investigated the potential association between exclusive breastfeeding and specific maternal and neonatal characteristics. It was conducted within the context of consistent and ongoing care provided by clinic midwives to all mothers, under the guidance of a research midwife who ensured that both Roma and non-Roma women received the same high level of care. Care was provided in a baby-friendly hospital environment, where standard practices included initiating breastfeeding within the first half-hour after birth and ensuring the rooming-in of newborns with their mothers. In our study, midwives provided equal support to all women, regardless of ethnicity, facilitating breastfeeding within the first hour after birth and implementing rooming-in immediately following delivery.

Data were analyzed using IBM SPSS Statistics version 26. The results of the exclusive breastfeeding rates among the Roma and non-Roma groups are presented in terms of frequencies and percentages. An independent samples t-test was performed to assess whether the exclusive breastfeeding rates differed significantly between Roma and non-Roma mothers. A Chi-square test was used to assess whether exposure to specific risk factors (RFs) was associated with exclusive breastfeeding as the outcome. Both Chi-square tests and logistic regression analyses were employed for data analysis. Multiple regression analysis was used to assess the statistical significance of additional factors that might also influence exclusive breastfeeding.

Factors investigated for their potential impact on exclusive breastfeeding included the mother’s occupation (housewife, unemployed, private employee, public employee, self-employed, welfare recipient), economic status (difficult, moderate, good, very good, excellent), age (18–35 years, 35–42 years, >42 years), parity (primiparous/multiparous), smoking status (smokers, non-smokers, or quit during pregnancy), type of delivery (vaginal or cesarean), and pregnancy outcome (complicated or uncomplicated). Notably, the mothers self-reported their economic status as difficult, moderate, good, very good, or excellent, meaning the classification was not based on actual income data. We also examined factors related to the newborn that could potentially influence exclusive breastfeeding, such as the infant’s gender (male or female) and birth weight (<2500 g or ≥2500 g).

The analyses were presented in two separate tables: one for Roma mothers and one for non-Roma mothers. In both, the dependent variable was the practice of exclusive breastfeeding, while the independent variables included all the maternal and neonatal factors mentioned earlier, which were considered potential influencers of exclusive breastfeeding. Data on maternal and neonatal characteristics, educational level, and socioeconomic status were collected through personal questionnaires. The analysis of each table predicted the likelihood of each independent variable influencing exclusive breastfeeding. The *p*-value was used to assess the statistical significance of each independent variable, with *p*-values (*p*) ≤ 0.05 being considered statistically significant.

## 3. Results

Our study included 53 Roma mothers and 196 non-Roma mothers. The prevalence of exclusive breastfeeding among Roma mothers was 19.2%, while among non-Roma mothers, it was 27%.

Among the non-Roma population, maternal occupation was found to significantly influence exclusive breastfeeding rates (*p* = 0.026). Specifically, housewives, unemployed mothers, and those receiving government benefits were more likely to exclusively breastfeed their infants compared to mothers working in the private sector, public sector employees, and self-employed mothers. Additionally, pregnancy outcome appeared to impact exclusive breastfeeding rates among non-Roma mothers (*p* = 0.042) (Table 1). Women who had an uncomplicated pregnancy were found to exclusively breastfeed their newborns at twice the rate compared to those who experienced complications during pregnancy. Furthermore, smoking was found to significantly influence the decision to exclusively breastfeed among non-Roma mothers (*p* = 0.043), whereas it appeared to have no effect on the decision among Roma mothers (*p* = 0.757). All other maternal and neonatal factors we investigated did not show a significant association with exclusive breastfeeding (Table 1).

For Roma mothers, economic status was found to influence exclusive breastfeeding rates (*p* = 0.034). Additionally, primiparous mothers were more likely to breastfeed their newborns exclusively compared to multiparous mothers (*p* = 0.026). Pregnancy outcome also significantly impacted the success of exclusive breastfeeding (*p* = 0.037) (Table 2).

## 4. Discussion

The primary aim of this study was to enhance healthcare service delivery and reduce maternity inequalities within marginalized groups, particularly among the Roma. Additionally, this research aimed to expand our understanding of effective maternity planning for these populations. While numerous studies globally investigate factors affecting breastfeeding, there remains a significant scarcity of research specifically focused on these marginalized groups.

Our study indicates that Roma mothers are less likely to exclusively breastfeed their infants compared to non-Roma mothers, with rates of 19.2% and 27%, respectively. This difference is statistically significant and aligns with findings from other countries, such as the study by Stamenković et al. [15] in Serbia, which also highlighted significant disparities in breastfeeding practices between Roma and non-Roma populations. Recent findings by Borja Herrero et al. [12] also show similar trends in breastfeeding rates among Roma communities in Spain, indicating a systemic issue across various regions.

In Greece, where overall breastfeeding rates are already low [16], the challenges faced by Roma women—such as socio-economic difficulties, cultural norms, and limited access to healthcare services—further exacerbate this disparity. Other marginalized minorities, such as immigrant communities [17] and low-income groups [18], also face comparable breastfeeding challenges, highlighting the need for tailored interventions.

Notably, our study also examined the impact of smoking on mothers’ decisions to breastfeed exclusively. We found that, among non-Roma mothers, smoking significantly influenced exclusive breastfeeding decisions (*p* = 0.043), whereas, for Roma mothers, the effect of smoking was negligible (*p* = 0.757). This discrepancy aligns with findings from other studies indicating that smoking can adversely affect breastfeeding practices. For instance, a study conducted by Cohen et al. [19] found that mothers who smoked were less likely to initiate and maintain breastfeeding compared to non-smokers. A similar trend has been observed in Greece, where research by Spyrakou et al. [20] provided evidence that lower maternal education, maternal smoking during pregnancy, cesarean section, prematurity, lower birth weight, and higher birth order were all negatively associated with breastfeeding initiation.

To date, there has been a limited amount of research in Greece specifically examining the impact of smoking on breastfeeding practices among Roma mothers. This gap in the literature highlights the need for targeted studies to understand how smoking may affect breastfeeding decisions within this community. Without such research, it is difficult to draw conclusions about the unique challenges faced by Roma mothers in relation to smoking and breastfeeding. Addressing this gap is essential for developing effective interventions that cater to the specific needs of Roma mothers and improve breastfeeding rates in this marginalized population. If future studies demonstrate that smoking negatively affects exclusive breastfeeding among Roma mothers, this suggests a need for tailored smoking cessation interventions specifically designed for Roma mothers that take into account cultural and socioeconomic factors.

Research indicates that culturally sensitive support systems, such as peer counseling programs [21] and community-based education initiatives [22], can significantly enhance breastfeeding rates in these populations. Furthermore, addressing systemic barriers—such as access to healthcare and socio-economic disparities—is crucial for developing effective breastfeeding support strategies [6,23]. These findings emphasize the need for interventions that not only consider cultural beliefs but also the unique challenges faced by these communities.

A significant finding from our study is that economic status markedly influences exclusive breastfeeding rates among Roma mothers (*p* = 0.034). Moreover, primiparous women are more likely to exclusively breastfeed compared to multiparous women (*p* = 0.026). Furthermore, pregnancy outcomes—such as preeclampsia, other hypertensive disorders, and gestational diabetes—were examined, as these complications significantly impacted breastfeeding patterns. Women with uncomplicated pregnancies were more likely to breastfeed exclusively compared to those experiencing complications (*p* = 0.037). These findings suggest that targeted interventions addressing economic and educational disparities, along with better management of pregnancy-related complications, could meaningfully improve breastfeeding rates within this community.

Despite receiving the same level of care from midwives and giving birth in a baby-friendly hospital—where all women were encouraged to breastfeed within the first half-hour and rooming-in was practiced—these differences in breastfeeding rates persisted. This finding underscores the influence of socio-cultural and economic factors, which may outweigh the benefits of standardized care protocols, highlighting the need for culturally tailored approaches to breastfeeding support. Studies focusing on African American and Hispanic communities similarly highlight the impact of cultural beliefs and systemic barriers on breastfeeding practices [24,25,26]. In this context, it is crucial to emphasize the importance of providing adequate lactation support to breastfeeding women. During their stay in the maternity ward, lactation support was provided through counseling sessions in a mother- and child-friendly hospital. Specifically, healthcare providers—including midwives—were trained to assist mothers in initiating lactation and addressing challenges they faced. The researcher, a midwife working at the hospital, offered daily support to breastfeeding women, ensuring that the breastfeeding protocol was consistently followed for both Roma and non-Roma groups. This collaboration aimed to prevent deviations from the established breastfeeding practices.

Breast pumps were available for mothers needing assistance with expressing milk, and midwives provided information on their usage. Protocols were established to support breastfeeding, including immediate skin-to-skin contact after birth and guidance on positioning and latching. These protocols align with international breastfeeding standards and aim to facilitate successful breastfeeding practices.

Furthermore, exploring the reasons some women opted for formula feeding is essential. Common reasons included a perceived insufficient milk supply, difficulties with latching, and a lack of confidence in breastfeeding. Understanding these factors can inform targeted interventions and enhance lactation support, particularly for women from ethnic minorities.

However, our study has several limitations that must be acknowledged. First, as a cross-sectional study, it cannot establish causal relationships between the examined factors and exclusive breastfeeding. Furthermore, our research focused on breastfeeding practices only up to discharge from the hospital, unlike studies such as that of Stamenković et al. [15], which examined breastfeeding prevalence up to six months postpartum. This limitation may mean that our findings do not fully capture the long-term breastfeeding practices of the study population. Additionally, our study did not assess certain factors that could have influenced breastfeeding, such as the mother’s desire for pregnancy, history of previous abortions, or participation in childbirth preparation programs. These factors, identified as significant in other studies, could have provided deeper insights into the breastfeeding behaviors of the population studied.

## 5. Conclusions

Promoting breastfeeding among Roma mothers necessitates a coordinated effort involving government agencies, healthcare professionals, and the Roma community itself. The findings of our study emphasize the need for culturally sensitive interventions that address the specific challenges faced by Roma women. These interventions should include targeted support from specialists such as midwives and lactation consultants, along with the development of educational programs tailored to the unique cultural and socio-economic context of the Roma community. Furthermore, targeted research is essential to explore long-term breastfeeding practices among Roma mothers and to assess the impact of smoking on these practices, as this could uncover additional barriers to exclusive breastfeeding. Future studies should investigate the socio-economic determinants of breastfeeding and how community engagement can enhance health outcomes. Moreover, incorporating childbirth preparation programs that specifically address the needs and concerns of Roma mothers could be particularly beneficial in increasing breastfeeding rates and improving maternal and infant health outcomes.

Despite the uniform care provided in a baby-friendly hospital environment—where all women were encouraged to initiate breastfeeding shortly after birth and practiced rooming-in—the disparities in breastfeeding rates between Roma and non-Roma mothers underscore the need to address underlying socio-cultural factors. This finding suggests that enhancing breastfeeding requires more than just standardized care; it necessitates a comprehensive and culturally responsive approach.

## Figures and Tables

**Table 1:** Maternal and neonatal risk factors potentially influencing exclusive breastfeeding among non-Roma mothers.

	Neonatal Nutrition				*p*-Value
Maternal_Age	Exclusive Breastfeeding	Breastfeeding and Formula	Formula	Total	0.313
18–35	43	88	22	153	
35–42	7	19	9	35	
>42	3	5	0	8	
Total	53	112	31	196	
Mother’s profession	Exclusive breastfeeding	Breastfeeding and formula	Formula	Total	0.026
Housewife	15	31	15	61	
Unemployed	13	37	4	54	
Private employee	2	0	0	2	
Self-employed	6	2	1	9	
Public employee	2	5	1	8	
Welfare recipient	15	37	10	62	
Total	53	112	31	196	
Pregnancy outcome	Exclusive breastfeeding	Breastfeeding and formula	Formula	Total	0.042
Complicated	18	32	16	66	
Uncomplicated	36	80	14	130	
Total	54	112	30	196	
Parity	Exclusive breastfeeding	Breastfeeding and formula	Formula	Total	0.14
Primiparous	27	68	15	110	
Multiparous	26	44	16	86	
Total	53	112	31	196	
Economic status	Exclusive breastfeeding	Breastfeeding and formula	Formula	Total	0.663
Difficult	11	30	7	48	
Moderate	20	45	15	80	
Good	21	35	8	64	
Very good	1	1	0	2	
Excellent	0	1	1	2	
Total	53	112	31	196	
Type of delivery	Exclusive breastfeeding	Breastfeeding and formula	Formula	Total	0.248
Vaginal birth	25	43	9	77	
Cesarean section	28	69	22	119	
Total	53	112	31	196	
Birth weight	Exclusive breastfeeding	Breastfeeding and formula	Formula	Total	0.511
<2500 g	6	10	5	21	
≥2500 g	47	102	26	175	
Total	53	112	31	196	
Infant’s gender	Exclusive breastfeeding	Breastfeeding and formula	Formula	Total	0.069
Male	21	63	17	101	
Female	33	49	13	95	
Total	54	112	30	196	
Smoking	Exclusive breastfeeding	Breastfeeding and formula	Formula	Total	0.043
Smokers	13	50	16	79	
Non-smokers	33	42	10	85	
Quitters	7	20	5	32	
Total	53	112	31	196	

**Table 2:** Maternal and neonatal risk factors potentially influencing exclusive breastfeeding among Roma mothers.

	Neonatal Nutrition				*p*-Value
Maternal_Age	Exclusive Breastfeeding	Breastfeeding and Formula	Formula	Total	0.706
18–35	9	25	8	42	
35–42	1	7	2	10	
>42	0	0	0	0	
Total	10	32	10	52	
Mother’s profession	Exclusive breastfeeding	Breastfeeding and formula	Formula	Total	0.397
Housewife	5	11	4	20	
Unemployed	2	15	3	20	
Private employee	0	0	0	0	
Self-employed	0	0	0	0	
Public employee	0	0	0	0	
Welfare recipient	3	6	3	12	
Total	10	32	10	52	
Pregnancy outcome	Exclusive breastfeeding	Breastfeeding and formula	Formula	Total	0.037
Complicated	6	6	4	16	
Uncomplicated	4	26	6	36	
Total	10	32	10	52	
Parity	Exclusive breastfeeding	Breastfeeding and formula	Formula	Total	0.026
Primiparous	5	20	3	28	
Multiparous	5	12	7	24	
Total	10	32	10	52	
Economic status	Exclusive breastfeeding	Breastfeeding and formula	Formula	Total	0.034
Difficult	2	9	2	13	
Moderate	6	13	5	24	
Good	1	10	2	13	
Very good	1	0	1	2	
Excellent	0	0	0	0	
Total	10	32	10	52	
Type of delivery	Exclusive breastfeeding	Breastfeeding and formula	Formula	Total	0.382
Vaginal birth	6	16	3	25	
Cesarean section	4	16	7	27	
Total	10	32	10	52	
Birth weight	Exclusive breastfeeding	Breastfeeding and formula	Formula	Total	0.207
<2500 g	1	1	2	4	
≥2500 g	9	31	8	48	
Total	10	32	10	52	
Infant’s gender	Exclusive breastfeeding	Breastfeeding and formula	Formula	Total	0.965
Male	6	18	6	30	
Female	4	14	4	22	
Total	10	32	10	52	
Smoking	Exclusive breastfeeding	Breastfeeding and formula	Formula	Total	0.757
Smokers	4	13	5	22	
Non-smokers	2	12	2	16	
Quitters	4	7	3	14	
Total	10	32	10	52	
